# Supplementary material for: TGF-β induces ST2 and programs ILC2 development
Source: Nat Commun. 2020 Jan 7;11:35. doi: 10.1038/s41467-019-13734-w (PMC6946674; doi:10.1038/s41467-019-13734-w)
Supplement: Supplementary file 1 — Supplementary Information [file 41467_2019_13734_MOESM1_ESM.pdf]

Supplementary figures and tables

TGF-β induces ST2 and programs ILC2 development

Li Wang[1,2*], Jun Tang[1,2,3*], Xia Yang[1,2*], Peter Zanvit[1*], Kairong Cui[4], Wai Lim Wang[4], Wenwen Jin[1], Dunfang Zhang[1], Nathan Goldberg[1], Alexander Cain[1], Bing Ni[2] , Keji Zhao[4], Yuzhang Wu[2], WanJun Chen[1]

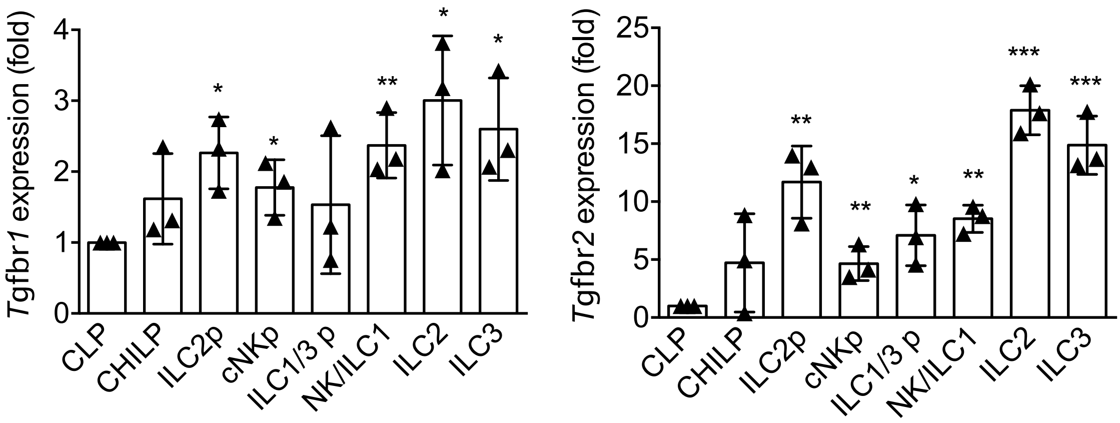

Supplementary Figure 1. *Tgfbr1* and *Tgfbr2* are expressed in ILC precursors and mature ILC cells.

Quantitative RT-PCR analysis of *Tgfbr1* and *Tgfbr2* expression in indicated cell populations isolated by cell sorting; results were normalized to *Hprt*. Data are pooled from 3 independent experiments and are presented as mean ± SEM (* $p<0.05$, ** $p<0.01$, *** $p<0.001$)

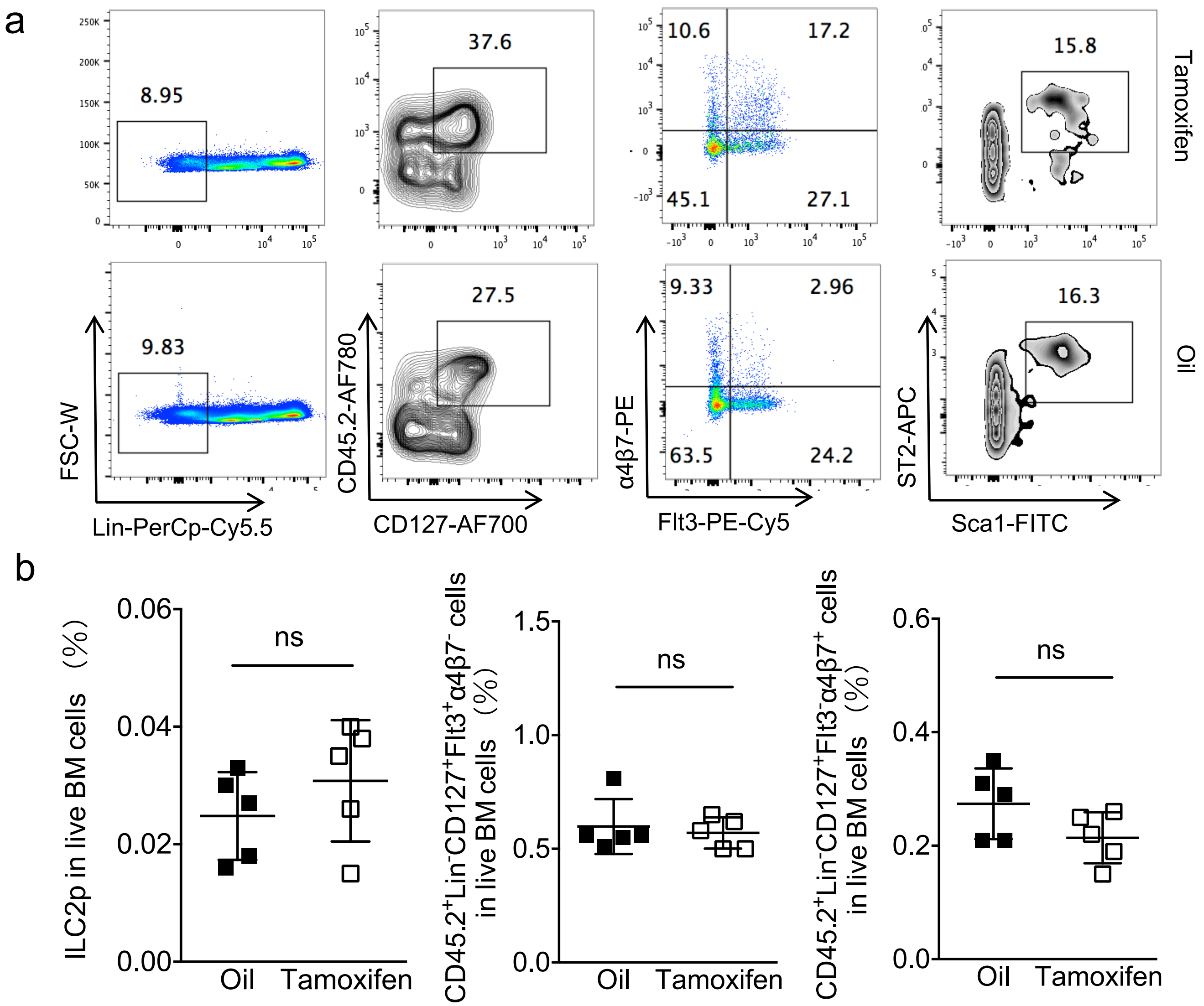

Supplementary Figure 2. No effect of Tamoxifen treatment on the bone marrow composition of *Tgfbr2*$^{f/f}$ER-Cre^{+} mice.

(a) Flow cytometry analysis of the frequency of CLP (CD45.2^{+}Lin^{-}CD127^{+}Fit3^{+}α4β7^{-}), ILC-committed progenitor (CD45.2^{+}Lin^{-}CD127^{+}Fit3^{-}α4β7^{+}) as well as ILC2 lineage-committed precursor (ILC2p) (CD45.2^{+}Lin^{-}CD127^{+}Fit3^{-}α4β7^{+}Sca1^{+}ST2^{+}) in bone marrow of oil or tamoxifen-treated *Tgfbr2*$^{f/f}$ER-Cre^{+} mice. Numbers indicate percentage of gated populations.

(b) Summarizing data showing frequency of ILC2p cells, CLP and ILC-committed progenitors in the bone marrow (n=5 mice per group). Significance was determined by Student's t-test (ns: no significance).

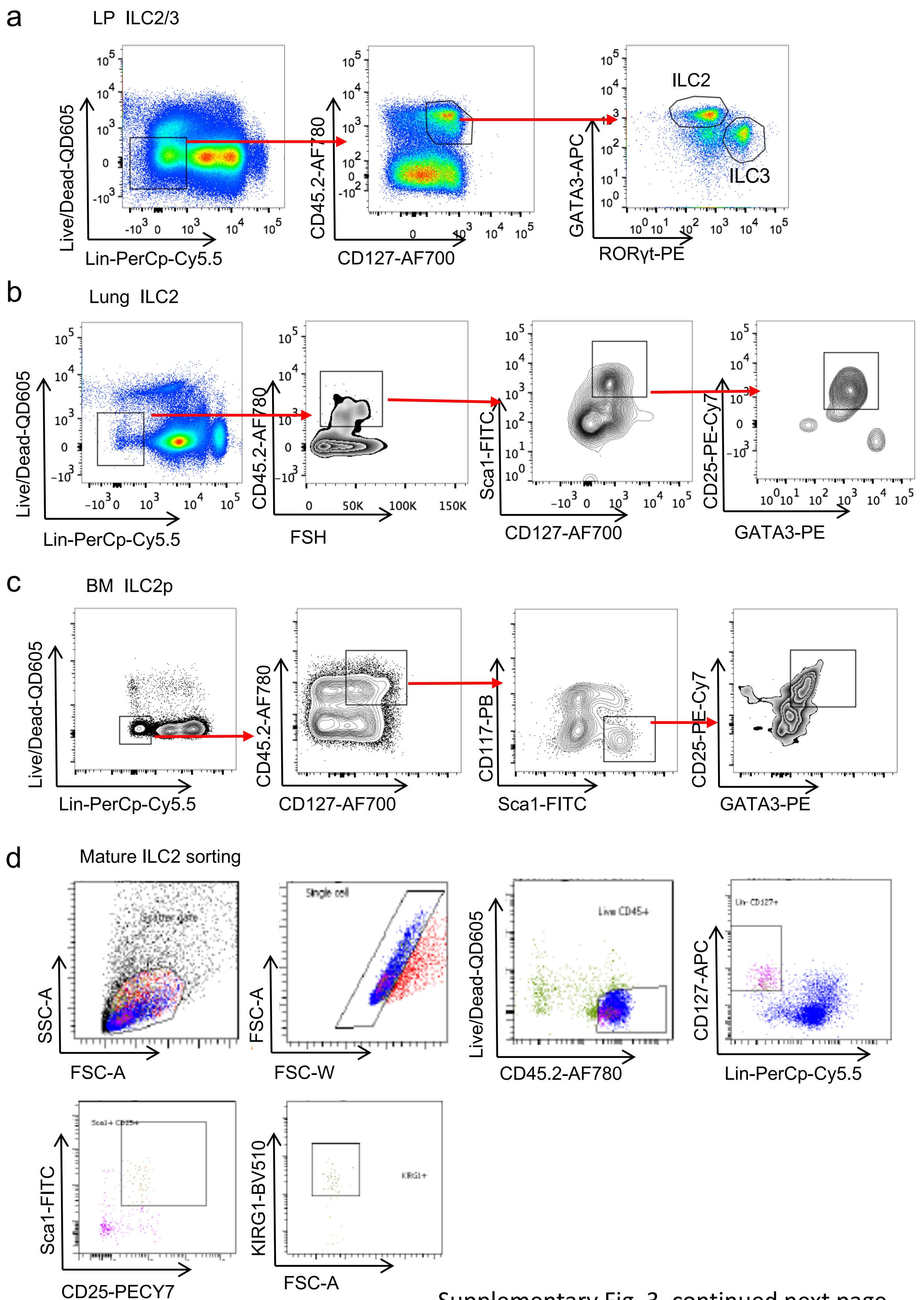

Supplementary Fig. 3, continued next page

e

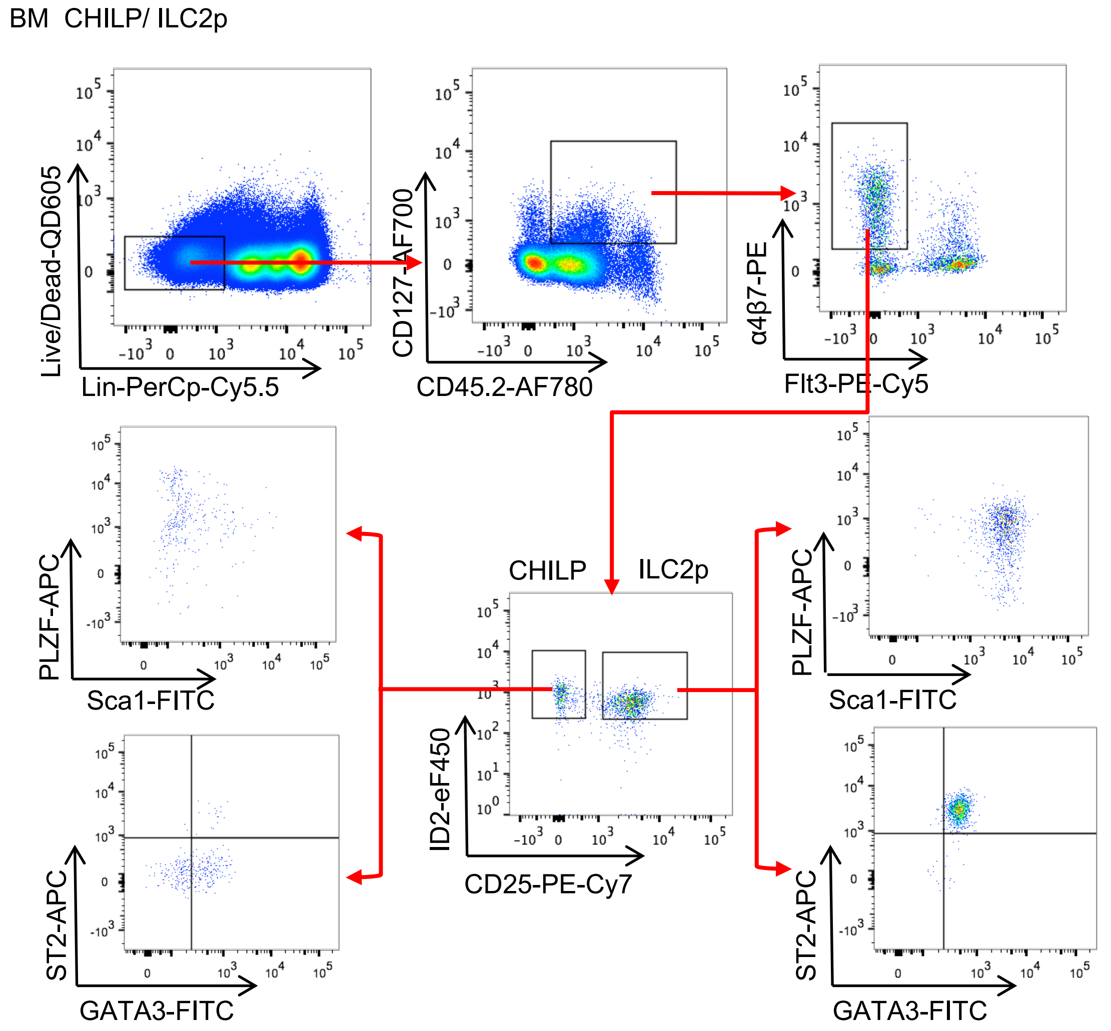

Supplementary Figure 3 Gating strategies for identification or sorting of lamina propria or bone marrow ILC2/ILC3 and ILC2p cells

Gating strategy for identifying: (a) $CD45.2^+Lin^-CD127^+GATA3^+$ ILC2s and $CD45.2^+Lin^-CD127^+ROR\gamma t^+$ ILC3s in the lamina propria (LP), (b) $CD45.2^+Lin^-CD127^+Sca1^+CD25^+GATA3^+$ ILC2 in the lung and (c) $CD45.2^+Lin^-CD127^+$ $CD117^-Sca1^+CD25^+GATA3^+$ ILC2-lineage-committed precursor (ILC2p) in the bone marrow (BM) by flow cytometry. (d) Gating strategy for sorting of the mature peripheral ILC2 cells ($CD45.2^+Lin^-Sca1^+CD25^+KLRG1^+$) (e) Gating strategy for identifying and sorting CHILP and ILC2p in the BM by flow cytometry.

Supplementary Figure 4

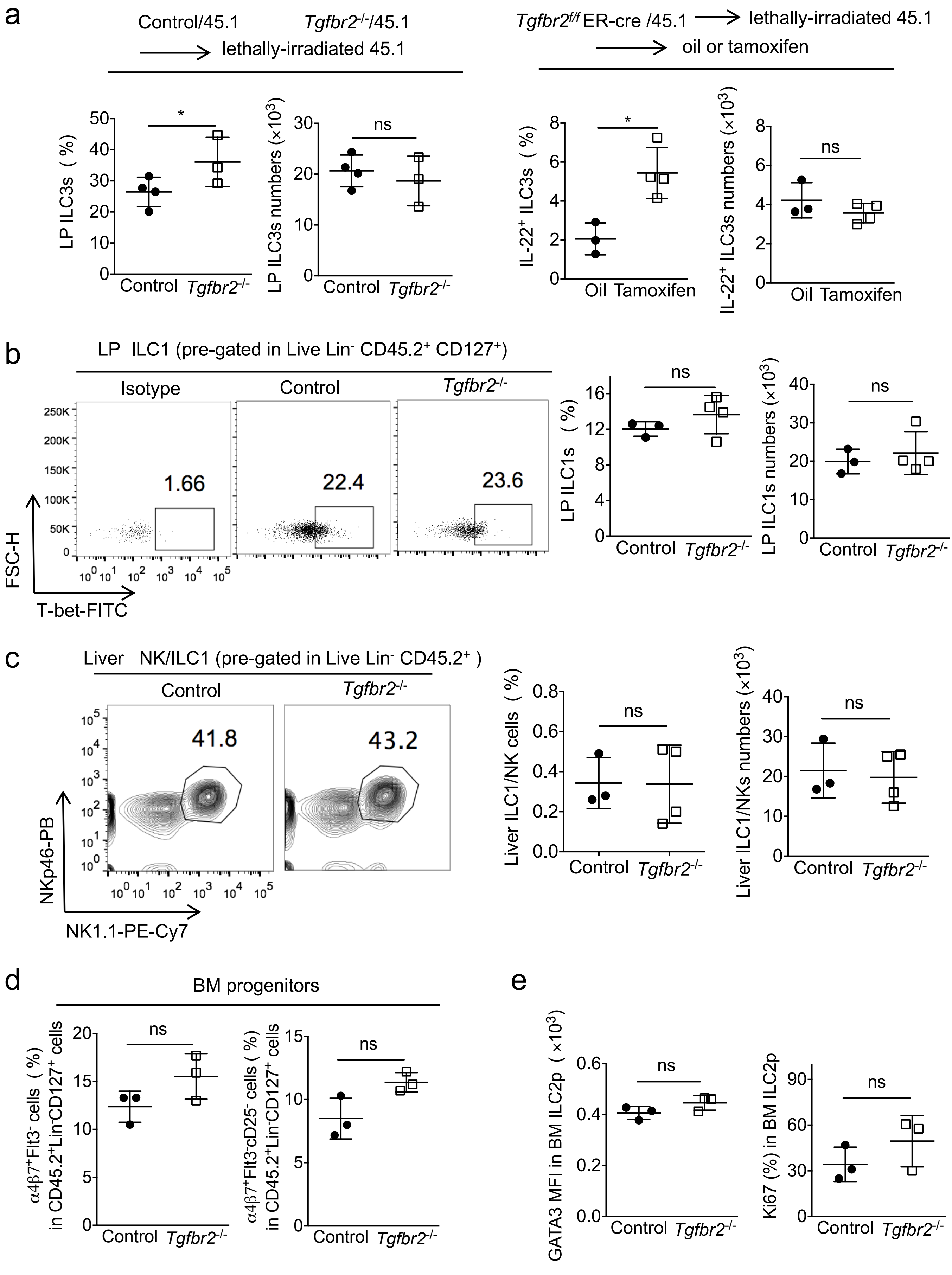

Supplementary Figure 4. TβR2 deletion has no effect on ILC3 and ILC1/NK cells in the periphery or the upstream of ILC2p in the BM.

(a) Summarized frequency and total numbers of CD45.2^{+} ILC3 cells in the LP of Control/CD45.1 and *Tgfbr2*$^{-/-}$/CD45.1 BM chimeras, and *Tgfbr2*$^{f/f}$ER-Cre^{+}/45.1 BM chimeras post-treated with tamoxifen or oil (n=3-4 mice per group).

(b) Representative FACS plot and summarized frequency and total number of of Tbet^{+} ILC1s among CD45.2^{+}Lin^{-}CD127^{+} cells in the LP from Control/CD45.1 and *Tgfbr2*$^{-/-}$/ CD45.1 BM chimeras (n=3-4 mice per group).

(c) Flow cytometry profiles and summary of the frequency and absolute number of NK1.1^{+}NKp46^{+}cells among CD45.2^{+}Lin^{-} cells in the liver from Control/CD45.1 and *Tgfbr2*$^{-/-}$/CD45.1 BM chimeras (n=3-4 mice per group).

(d) Summary of the frequency of α4β7^{+}Flt3^{-} progenitor cells and α4β7^{+}Flt3^{-}CD25^{-} progenitor cells among CD45.2^{+}Lin^{-}CD127^{+} cells in the BM of Control/CD45.1 and *Tgfbr2*$^{-/-}$/CD45.1 BM chimeras (n=3 mice per group).

(e) MFI of GATA3 and frequency of Ki67 expression in ILC2p in the BM of Control/CD45.1 and *Tgfbr2*$^{-/-}$/CD45.1 BM chimeras (n=3 mice per group).

All data are representative of two independent experiments. Data are represented as mean ± SD; *p<0.05, ns: no significance (Student's t-test).

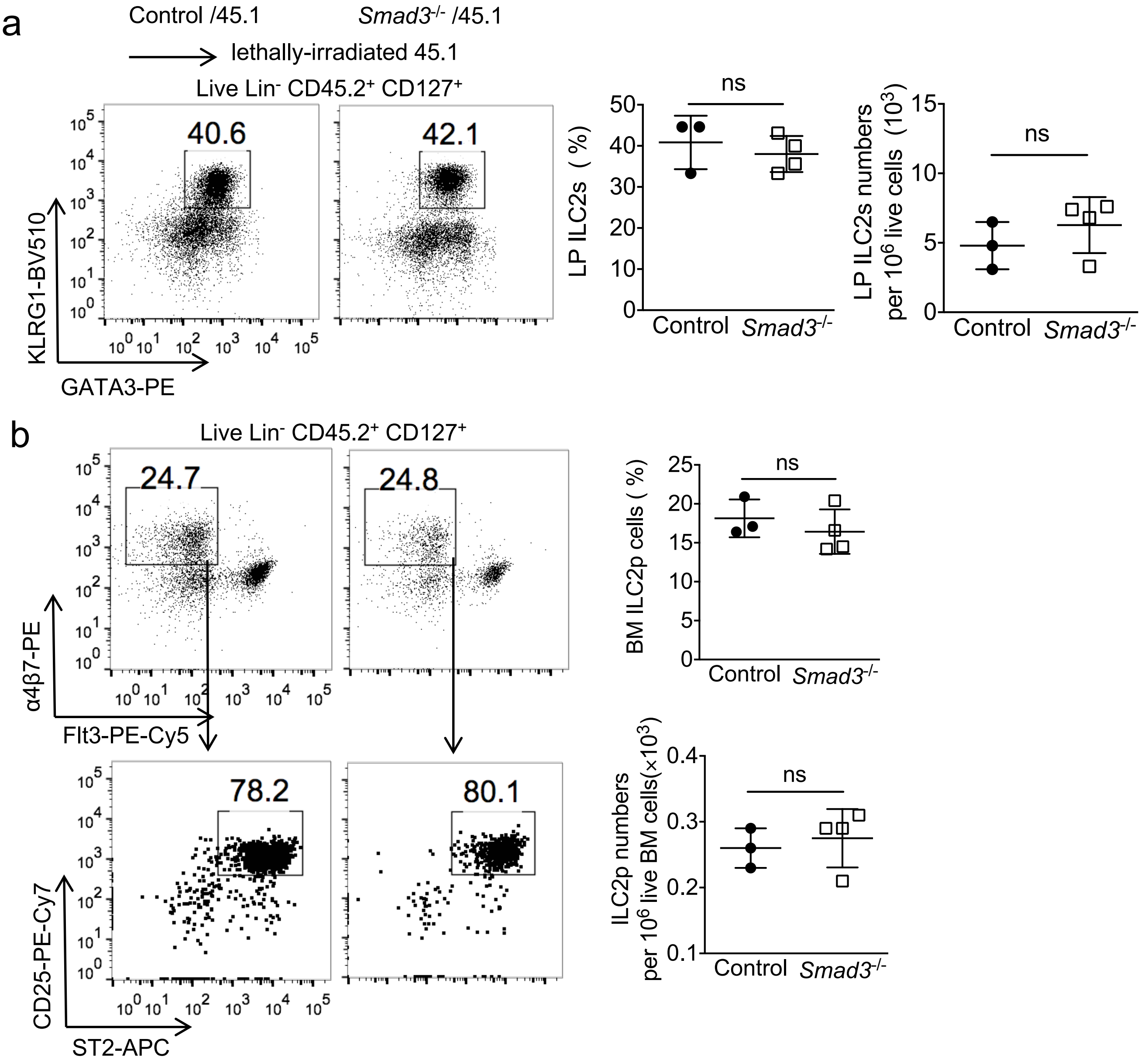

Supplementary Figure 5. Intrinsic SMAD3 deficiency has no effect on the ILC2 generation.

(a) Representative FACS plots and summarized frequency and total number of KLRG1$^+$GATA3$^+$ ILC2 cells among CD45.2$^+$Lin$^-$CD127$^+$ cells in the LP of Control/CD45.1 and *Smad3*$^{-/-}$/CD45.1 BM chimeras (n=3-4 mice per group).
(b) Representative FACS plots and summarized frequency and total number of $\alpha_4\beta_7$$^+$Flt3$^-$ CD25$^+$ST2$^+$ ILC2p cells among CD45.2$^+$Lin$^-$CD127$^+$ cells in the BM of Control/CD45.1 and *Smad3*$^{-/-}$/ /CD45.1 BM chimeras (n=3-4 mice per group).
Data are representative of two independent experiments and shown as mean ± SD; ns: no significance (Student's t-test).

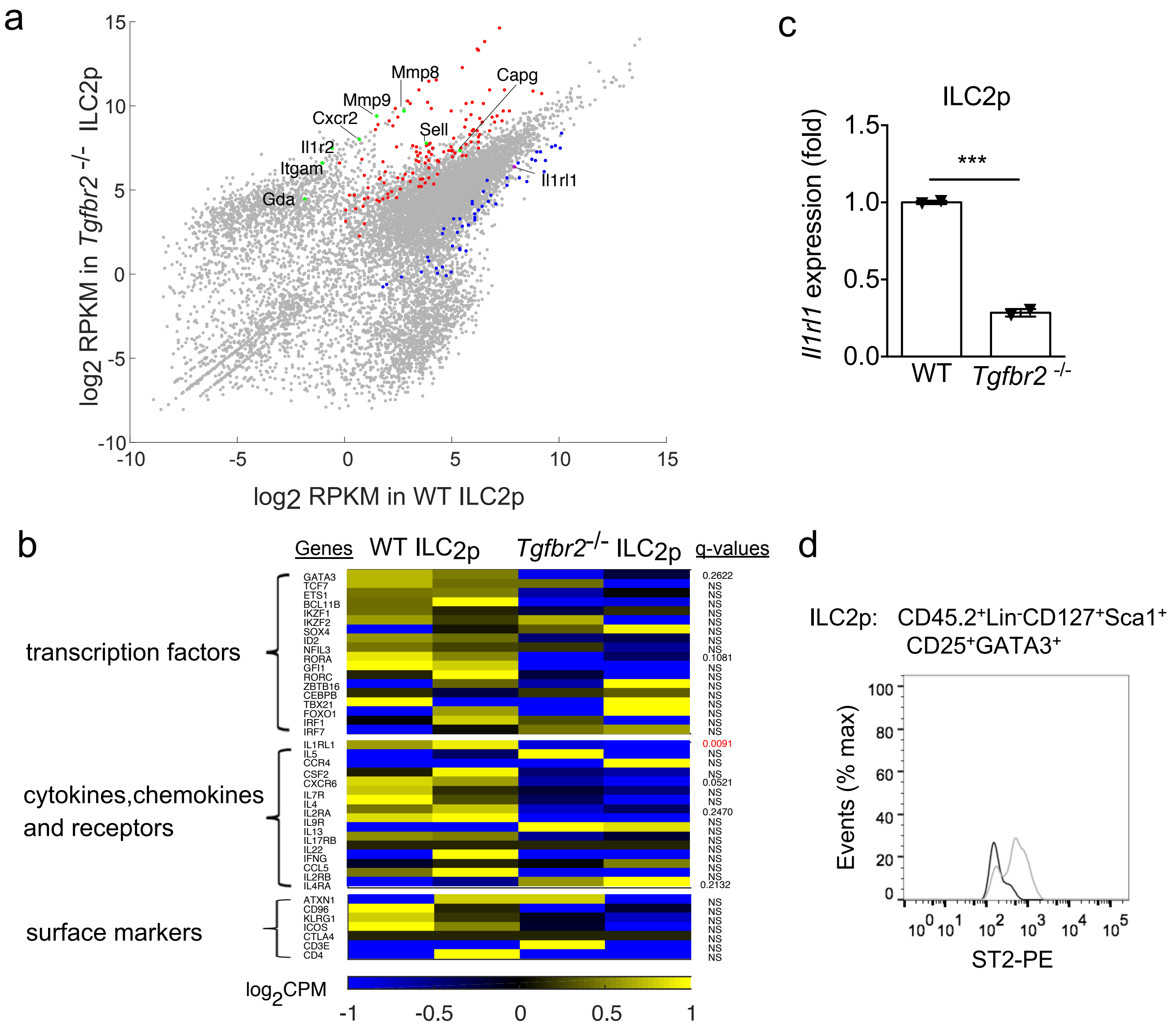

Supplementary Figure 6. RNA-seq analysis of global transcriptome in *Tgfbr2*$^{-/-}$/WT ILC2p. (a) RNA-seq analysis of sorted *Tgfbr2*$^{-/-}$ and WT ILC2p cells (CD45.2^{+}Lin^{-}Flt3^{-} α4β7^{+}CD25^{+}) in the BM of Control/CD45.1 and *Tgfbr2*$^{-/-}$/CD45.1 BM chimeras. Data are shown as a scatter plot of log2 of FPKM (fragments per kilo base of transcript per million mapped reads) from *Tgfbr2*$^{-/-}$ and WT ILC2p cells reconstituted for 6 weeks. Blue dots represent downregulated genes, and red dots represent upregulated genes in *Tgfbr2*$^{-/-}$ILC2p cells. Genes of interest are indicated and marked in purple (downregulated) and green (upregulated). (b) Heat map of the expression of select genes (left margin) in WT and *Tgfbr2*$^{-/-}$ ILC2p cells, grouped according to the function of their products (left margin). NS, not significant (q value, ≥ 0.2) (Student's t-test, Benjamini and Hochberg correction for multiple tests). (c) Quantitative RT-PCR analysis of the *Il1rl1* in the sorted *Tgfbr2*$^{-/-}$ ILC2p cells relative to WT ILC2p cells in BM. Data is representative of 2 independent experiments. Data are shown as mean ± SD; *** p<0.001(Student's t-test). (d) Representative FACS histogram of ST2 expression in WT (gray line) and *Tgfbr2*$^{-/-}$ ILC2p cells (black line).

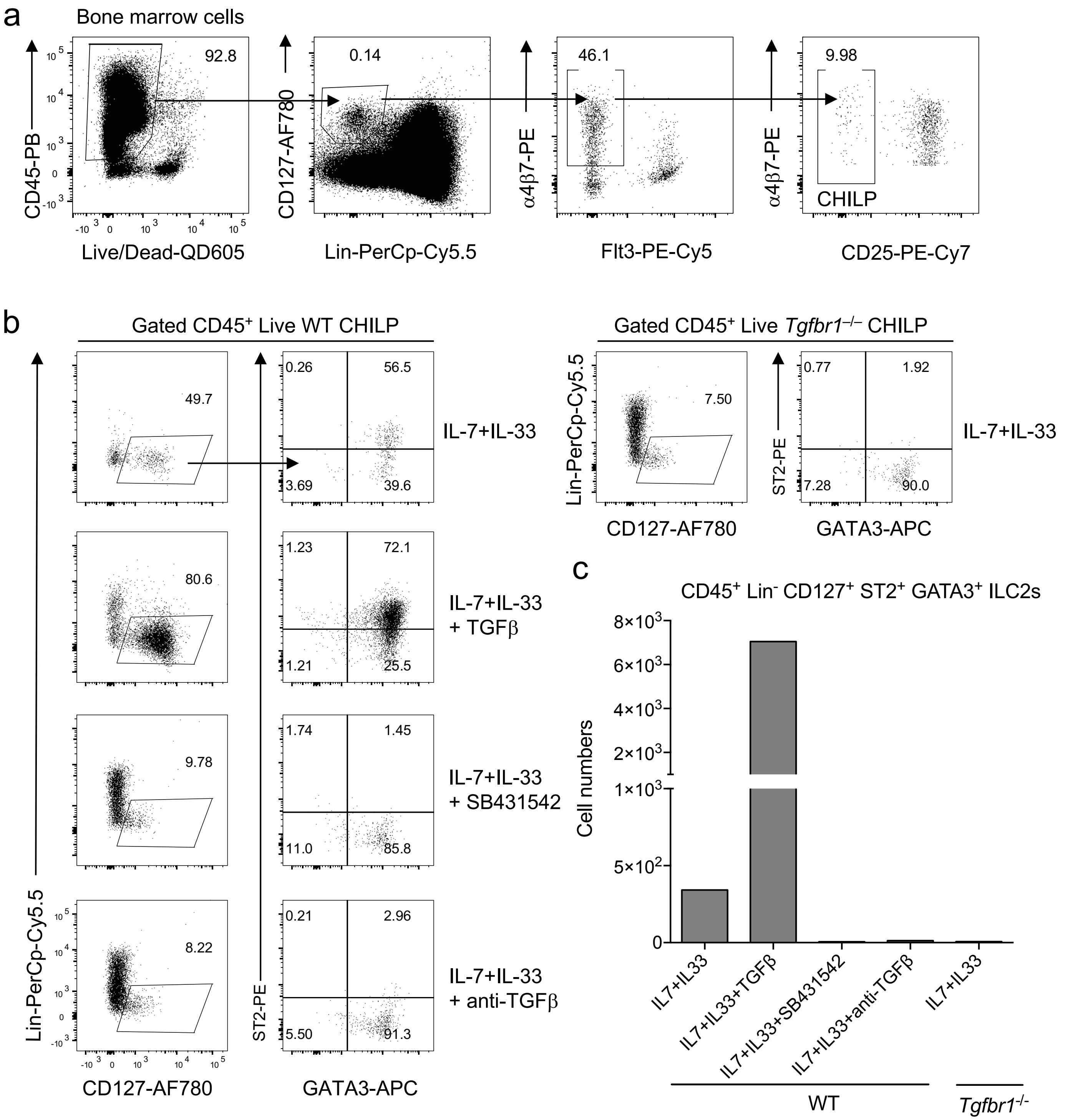

Supplementary Figure 7. TGF-β promotes the generation of ILC2 from CHILP in vitro

(a) Representative gating strategy for sorting of Live CD45$^+$CD127$^+$α4β7$^+$CD25$^-$ bone marrow CHILP cells. (b) Representative FACS plots are depicting frequencies of Live CD45$^+$Lin$^-$CD127$^+$ or Live CD45$^+$Lin$^-$CD127$^+$GATA3$^+$ST2$^+$ ILC2 cells respectively. WT or *Tgfbr1*$^{-/-}$ CHILP cells were co-cultured with OP9-DL1 cells in presence of IL-7 (20 ng/mL) and IL-33 (20 ng/mL) or TGF-β (2ng/mL) or TβR1 inhibitor SB431542 (5 μM) or anti-TGF-β (50 μg/mL) for 13 days and analyzed using flow cytometry. (c) Summarizing figure showing total number of CD45$^+$Lin$^-$CD127$^+$GATA3$^+$ST2$^+$ (ILC2) in CHILP cultures (day 13) of WT and *Tgfbr1*$^{-/-}$ cells. Data shown are representative of 2 (*Tgfbr1*$^{-/-}$ CHILP) and 3 (WT CHILP) independent experiments.

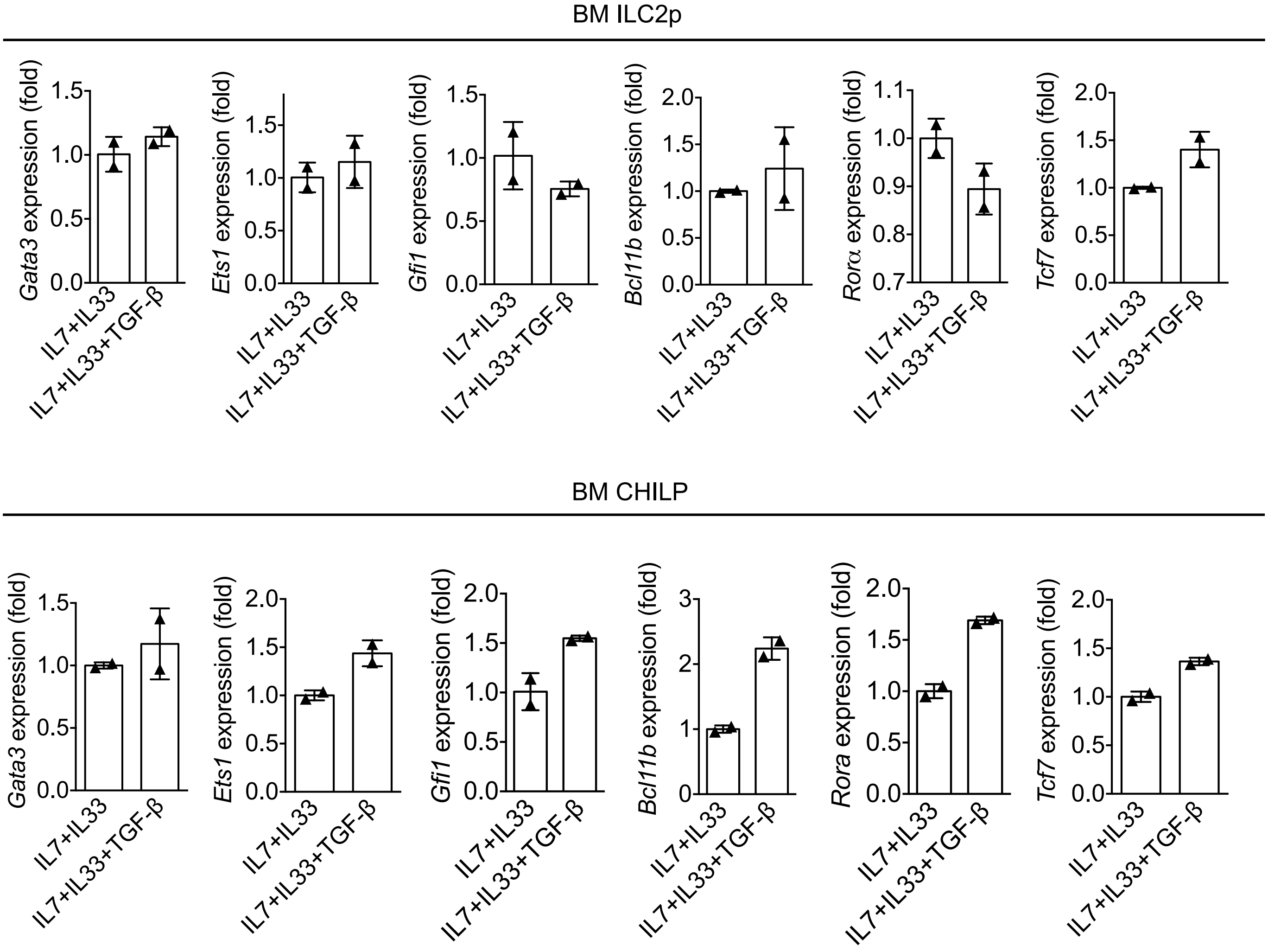

Supplementary Figure 8. Gene expression of bone marrow ILC2p and CHILP from cultures treated with/without TGF-β for 24 hours.

Data are representative of one out of 2 independent experiments. Data were analyzed using qPCR and shown as mean ± SD.

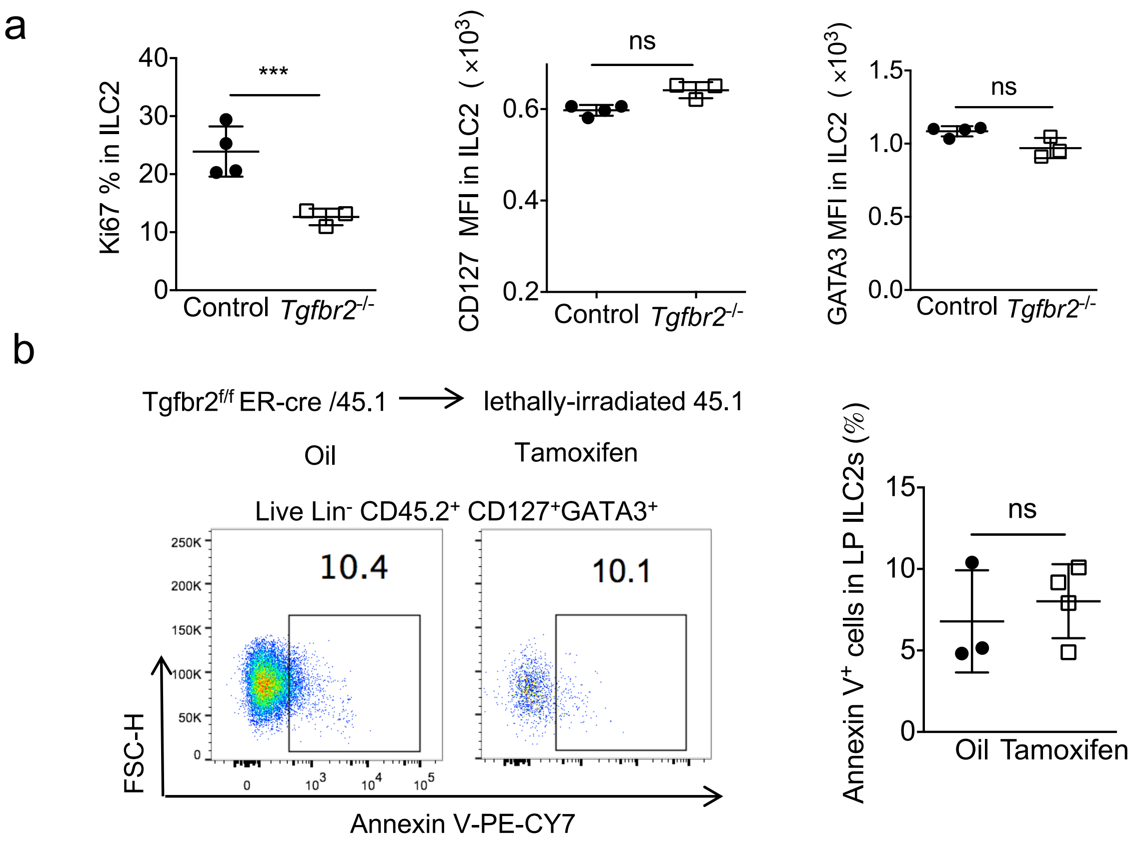

Supplementary Figure 9. TβR2 deletion decreases the proliferation of ILC2 cells.

(a) Frequency of Ki67 and MFI's of CD127 and GATA3 in mature CD45.2$^+$ ILC2 cells in the LP of Control/CD45.1 and *Tgfbr2*$^{-/-}$ CD45.1 BM chimeras (n=3-4 mice per group).

(b) Representative FACS plots showing Annexin-V expression on the surface of ILC2s in the LP of of *Tgfbr2*$^{f/f}$ER-Cre$^+$/45.1 BM chimeras post-treated with tamoxifen or oil (n=3-4 mice per group).

Data are representative of two independent experiments and presented as mean ± SD.

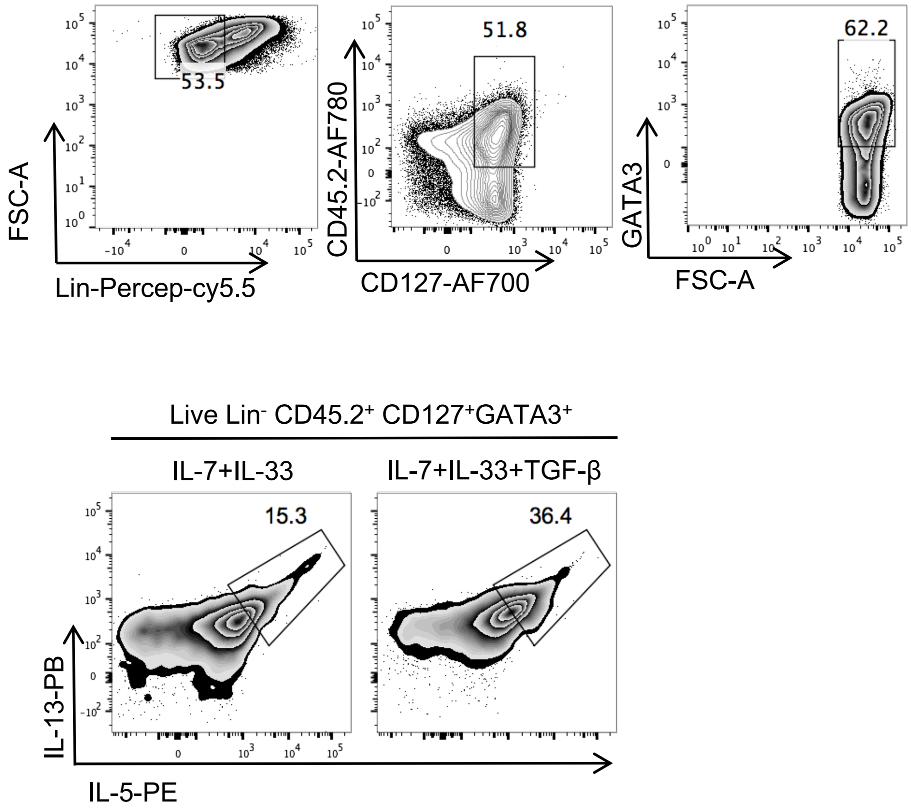

Supplementary Figure 10. TGF-β increases ILC2 cells production of IL-13 and IL-5 in *in vitro* culture.

Flow cytometry profiles of cells derived from sorted WT ILC2 precursors (ILC2p; Lin^{-} $CD127^{+}Flt3^{-}\alpha_4\beta_7{}^{+}CD25^{+}$) cultured with OP9-DL1 stromal cells plus IL-7 and IL-33, in the presence or absence of rhTGF-β1, and analyzed for the intracellular IL-13 and IL-5 expression from ILC2s (Live $CD45.2^{+}Lin^{-}$ $CD127^{+}GATA3^{+}$) stimulated with PMA plus ionomycin after 13 days of culture.

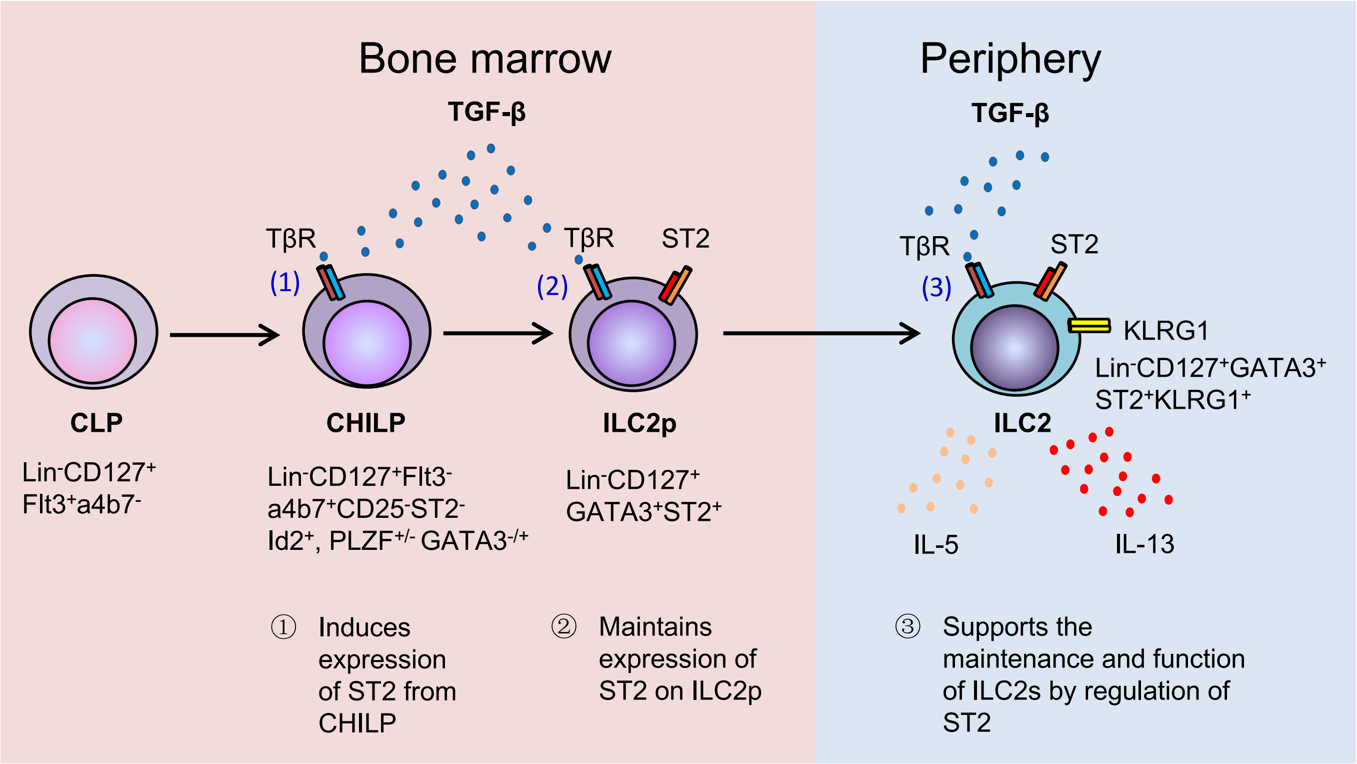

Supplementary Fig 11. A proposed model for the role of TGF-β in the development of ILC2 cells from their BM precursors.

(1) TGF-β signaling is essential for the induction of ST2 on CHILP cells and enhances the development and generation of ST2+ ILC2p cells

(2) Once ILC2p (ST2 positive) are generated, TGF-β signaling is needed to maintain ST2 expression at high level, which may regulate ILC2p differentiation/proliferation into ILC2;

(3) TGF-β signaling supports the maintenance and the function of mature ILC2 in the periphery by regulating their ST2 expression.

Supplementary Table 1: Surface markers for identification and sorting of the indicated cell subsets

**Name**	**Identifying markers for sorting**	**Cell subsets**
CLP	$CD45^{+}Lin^{-}CD127^{+}Flt3^{+}\alpha_4\beta_7^{-}$	T, B, ILC potential
$Lin^{-}\alpha_4\beta_7^{+}$ progenitors	$CD45^{+}Lin^{-}CD127^{+}Flt3^{-}\alpha_4\beta_7^{+}$	ILC potential
$Lin^{-}\alpha_4\beta_7^{+}CD25^{-}$ progenitors	$CD45^{+}Lin^{-}CD127^{+}Flt3^{-}\alpha_4\beta_7^{+}CD25^{-}$	Includes CHILP
$Lin^{-}\alpha_4\beta_7^{+}CD25^{+}$ progenitors	$CD45^{+}Lin^{-}CD127^{+}Flt3^{-}\alpha_4\beta_7^{+}CD25^{+}$	Contains ILC2 progenitor
ILC2p	$CD45^{+}Lin^{-}CD127^{+}Flt3^{-}\alpha_4\beta_7^{+}CD25^{+}Sca1^{+}CD117^{-}GATA3^{+}$	ILC2 progenitor
cNKp	$CD45^{+}Lin^{-}NK1.1^{+}NKp46^{+}CD127^{-}$	cNK precursor
ILC1/3p	$CD45^{+}Lin^{-}NK1.1^{+}NKp46^{+}CD127^{+}$	ILC1/3 precursor
Liver NK/ILC1	$CD45^{+}Lin^{-}NK1.1^{+}NKp46^{+}$	Mature NK and ILC1
LP ILC1	$CD45^{+}Lin^{-}CD127^{+}Tbet^{+}$	Mature ILC1
LP ILC2	$CD45^{+}Lin^{-}CD127^{+}GATA3^{+}$ (analysis) or $CD45^{+}Lin^{-}CD127^{+}Sca1^{+}CD25^{+}KIRG1^{+}$ (sorting)	Mature ILC2
LP ILC3	$CD45^{+}Lin^{-}CD127^{+}ROR\gamma^{+}$ (analysis) or $CD45^{+}Lin^{-}CD127^{+}CD117^{+}Sca1^{-}CD25^{-}$ (sorting)	Mature ILC3
Lung ILC2	$CD45^{+}Lin^{-}CD127^{+}Sca1^{+}CD25^{+}ST2^{+}GATA3^{+}$	Mature ILC2

Supplementary Table 2: Antibodies used in this study

Fluorescein	Name (clone)
Percep-cy5.5	CD3ε (145-2C11), CD5 (53-7.3), CD11b (M1/70), CD11c (N418), CD19 (1D3), B220 (RA3-6B2), Gr-1 (RB6-8C5), Nk1.1 (PK136), NKp46 (29A1.4), CD23 (MAR-1)
APC	CD25 (7D4), CD127 (A7R34), ST2 (RMST2-2), GATA3 (TWAJ), RORγt (B2D), Tbet (eBio4B107), IL-22 (IL22JOP), IL-13 (eBio13A)， PLZF (9E12)
PE	CD45 (C363.16A), α4β7 (DATK32), NK1.1, ST2 (RMST2-2), GATA3 (TWAJ), RORγt (B2D), Tbet (eBio4B107), IL-5 (TRFK5), IL-17 (eBio17B7),
AF-700	CD127 (A7R34), CD49b (DX5)
PE-CY7	CD25 (PC61.5), Annexin-V, NK1.1 (PK136)
PE-CY5	Flt3 (A2F10)
FITC (AF488)	CD45 (30-F11), CD45.2 (104), CD45.1 (A20), CD25 (eBio7D4), Sca1 (D7), CD27 (LG.3A10), PLZF (Mags.21F7)， GATA3 (TWAJ)
AF780	CD45.2 (104), CD127 (A7R34)
Pacific blue (eF450)	CD45.1 (A20), NKp46 (29A1.4), CD117 (2B8)， ID2 (ILCID2)
PE-ef610	Ki67 (SolA15)
BV510	KLRG-1 (MAFA)
QD605	Zombie yellow
	anti-CD16/CD32 (93)
