# Supplementary material for: TGF-β induces ST2 and programs ILC2 development
Source: Nat Commun. 2020 Jan 7;11:35. doi: 10.1038/s41467-019-13734-w (PMC6946674; doi:10.1038/s41467-019-13734-w)
Supplement: Supplementary file 3 — Reporting Summary [file 41467_2019_13734_MOESM3_ESM.pdf]

nature research

Corresponding author(s): Chen, Wanjun. NI-A25238B

Reporting Summary

Nature Research wishes to improve the reproducibility of the work that we publish. This form provides structure for consistency and transparency in reporting. For further information on Nature Research policies, see Authors & Referees and the Editorial Policy Checklist.

Statistical parameters

When statistical analyses are reported, confirm that the following items are present in the relevant location (e.g. figure legend, table legend, main text, or Methods section).

n/a	Confirmed	
☐	☒	The exact sample size (n) for each experimental group/condition, given as a discrete number and unit of measurement
☐	☒	An indication of whether measurements were taken from distinct samples or whether the same sample was measured repeatedly
☐	☒	The statistical test(s) used AND whether they are one- or two-sided<br>*Only common tests should be described solely by name; describe more complex techniques in the Methods section.*
☐	☒	A description of all covariates tested
☐	☒	A description of any assumptions or corrections, such as tests of normality and adjustment for multiple comparisons
☐	☒	A full description of the statistics including central tendency (e.g. means) or other basic estimates (e.g. regression coefficient) AND variation (e.g. standard deviation) or associated estimates of uncertainty (e.g. confidence intervals)
☐	☒	For null hypothesis testing, the test statistic (e.g. F, t, r) with confidence intervals, effect sizes, degrees of freedom and P value noted<br>*Give P values as exact values whenever suitable.*
☒	☐	For Bayesian analysis, information on the choice of priors and Markov chain Monte Carlo settings
☒	☐	For hierarchical and complex designs, identification of the appropriate level for tests and full reporting of outcomes
☒	☐	Estimates of effect sizes (e.g. Cohen's d, Pearson's r), indicating how they were calculated
☐	☒	Clearly defined error bars<br>*State explicitly what error bars represent (e.g. SD, SE, CI)*

Our web collection on statistics for biologists may be useful.

Software and code

Policy information about availability of computer code

Data collection	Flow cytometry measurments were performed on BD LSRII Fortessa instrument, data was collected via FACSDiva 8.0.1 Software.
Data analysis	GraphPad Prism 7, FlowJo 10, EdgeR, FACS Diva Softwares were used for data analysis. For additional details please refer to Methods section.

For manuscripts utilizing custom algorithms or software that are central to the research but not yet described in published literature, software must be made available to editors/reviewers upon request. We strongly encourage code deposition in a community repository (e.g. GitHub). See the Nature Research guidelines for submitting code & software for further information.

Data

Policy information about availability of data

All manuscripts must include a data availability statement. This statement should provide the following information, where applicable:

- Accession codes, unique identifiers, or web links for publicly available datasets
- A list of figures that have associated raw data
- A description of any restrictions on data availability

All data generated and supporting the findings of our study are available within the paper.

Field-specific reporting

Please select the best fit for your research. If you are not sure, read the appropriate sections before making your selection.

- [x] Life sciences
- [ ] Behavioural & social sciences
- [ ] Ecological, evolutionary & environmental sciences

For a reference copy of the document with all sections, see nature.com/authors/policies/ReportingSummary-flat.pdf

Life sciences study design

All studies must disclose on these points even when the disclosure is negative.

Sample size	No statistical methods were used to predetermine sample size. At minimum of 3 individual mice were used for all the experiments and assumed this would be required to recognize differences between genotypes or conditions. Additional details for each figure panel is included in the figure legends.
Data exclusions	No data were excluded.
Replication	Experiments were replicated at least twice as described throughout the manuscript and in the Methods. Additional information of the each figure panel is included in the figure legend.
Randomization	We did not use any randomization. All mice of same background, similar age and sex were used in all experiments.
Blinding	The investigators were not blinded to the identities of the samples because treatments and data collection were performed by the same people. All samples were analyzed at the same time under the same conditions.

Reporting for specific materials, systems and methods

Materials & experimental systems

n/a	Involved in the study	
x		Unique biological materials
	x	Antibodies
	x	Eukaryotic cell lines
x		Palaeontology
	x	Animals and other organisms
x		Human research participants

Methods

n/a	Involved in the study	
x		ChIP-seq
	x	Flow cytometry
x		MRI-based neuroimaging

Antibodies

Antibodies used	The following antibodies were used: CD3ε (145-2C11), CD5 (53-7.3), CD11b (M1/70), CD11c (N418), CD19 (1D3), B220 (RA3-6B2), Gr-1 (RB6-8C5), Nk1.1 (PK136), NKp46 (29A1.4), CD23 (MAR-1), CD25 (7D4), CD127 (A7R34), ST2 (RMST2-2), GATA3 (TWAJ), RORγt (B2D), Tbet (eBio4B107), IL-22 (IL22JOP), IL-13 (eBio13A), PLZF (9E12), CD45 (C363.16A), α4β7 (DATK32), NK1.1, IL-5 (TRFK5), IL-17 (eBio17B7), CD49b (DX5), CD25 (PC61.5), Annexin-V, NK1.1 (PK136), Flt3 (A2F10), CD45 (30-F11), CD45.2 (104), CD45.1 (A20), CD25 (eBio7D4), Sca1 (D7), PLZF (Mags.21F7), CD117 (2B8), ID2 (ILCID2), Ki67 (SolA15), KLRG-1 (MAFA). Live/dead cell were discriminated based on Zombie yellow staining (Biolegend). anti-CD16/CD32 (93) was used for FC receptor blocking.
Validation	All the antibodies used have undergone extensive validation by the manufacturer.

Eukaryotic cell lines

Policy information about cell lines

Cell line source(s)	OP9-DL1 cells were obtained from the Zúñiga-Pflücker laboratory.
Authentication	No authentication was performed.
Mycoplasma contamination	The OP9-DL1 cell line were negative for mycoplasma contamination.

Commonly misidentified lines
(See ICLAC register)

N/A

Animals and other organisms

Policy information about studies involving animals; ARRIVE guidelines recommended for reporting animal research

Laboratory animals	C57BL/6 and CD45.1 male and female mice were obtained from the Jackson Laboratory. Tgfbr2f/f ER-Cre+ mice (C57BL/6 background), Smad3–/– mice, Tak1f/f ER-Cre+ and Rag2–/– IL2rg–/– mice were bred under specific pathogen-free conditions in the animal facility of the National Institute of Dental and Craniofacial Research. 6-12 week old mice were used in this study.
Wild animals	No wild animals were used.
Field-collected samples	No field-collected samples were used.

Flow Cytometry

Plots

Confirm that:

- [x] The axis labels state the marker and fluorochrome used (e.g. CD4-FITC).
- [x] The axis scales are clearly visible. Include numbers along axes only for bottom left plot of group (a 'group' is an analysis of identical markers).
- [x] All plots are contour plots with outliers or pseudocolor plots.
- [x] A numerical value for number of cells or percentage (with statistics) is provided.

Methodology

Sample preparation	Small intestines and colons of mice were removed and opened longitudinally and were washed in PBS to remove the fecal contents. Intestines were cut into segments 2cm in length, then transferred into pre-warmed IEL isolation media (DMEM, 4%FBS, 5mM EDTA, and 0.145 mg/ml DTT) and incubated for 20 min at 37°C with stirring. The remaining intestinal tissue was washed three times with DMEM containing 2 mM EDTA to remove epithelial cells and fat tissue, and then was minced and incubated in DMEM digestion medium containing liberase (10 mg/ml) and 0.05% DNase for 30 min at 37°C with gentle shaking. Digested suspensions were filtered and washed in PBS. Whole lungs were minced and digested in DMEM (10% FBS) containing 1 mg/ml collagenase type IV (Gibco) and 0.5 mg/ml DNase I (Roche Diagnostics) for 20 min at 37°C with shaking at 100 rpm. The liver was thoroughly dissected and gently passed through a 200-gauge stainless steel mesh. Lymphocytes from lung, liver, and gut were all isolated using 30% Percoll solution in DMEM. Cells were then filtered with a 70-μm cell strainer and red blood cells were lysed. BM single-cell suspensions were prepared from mice femurs, tibias, and humerus by flushing the shaft with buffer using a syringe and a 25G needle. For Collection of BAL Fluid, mice were injected intranasally with 100μg HDM in 50μl PBS on days 0–2 and further euthanized on day 3. A midline incision was made to retract the skin and the connective tissue surrounding the trachea is carefully removed. A suture thread was placed underneath the trachea by carefully lifting it with tweezers. A small incision between the tracheal cartilage was made to inserta cannula, be careful not to sever the entire trachea. The cannula is secured in place using the suture thread by tying a knot around the portion of the trachea housing the cannula. A syringe containing 1 mL PBS supplemented with 0.5 mM EDTA was attached to the cannula. The lungs were subsequently washed by gently flushing. Afterwards, the syringe is uncoupled and the aspirated BAL fluid is collected in a tube. Repeat wash for two more times for a total of ~3 mL BAL fluid. BAL fluid are centrifuged at 4 °C 400 × g for 7 min and BAL cell pellets were resuspended for cell counting and were stained for flow cytometric applications. Surface staining was performed in PBS containing 2% fetal bovine serum (wt/vol) on ice. Intracellular staining was performed by using Foxp3/Transcriptional Factor Staining Buffer Set (00-5523; eBioscience).
Instrument	BD LSRII Fortessa
Software	BD FACSDiva 8.0.1 (BD Biosciences), FlowJo 10 (Tree Star)
Cell population abundance	Cell subsets were sorted on a BD FACS Aria III instrument. Post sort samples were analyzed on the same FACS Aria III machine indicating greater than 90% purity for sorted subsets.
Gating strategy	The gating strategy is provided in Supplementary figure 2. All ILC subsets and their progenitors were gated as: small/non-granular (FSClow/ SSClow), single (FSC-W/FSC-H and SSC-W/SSC-H), live (Zombie-negative), leukocytes (CD45+). The boundaries between positive and negative populations (e.g. CD127, ST2, Gata3, RORgt, Tbet,) were defined based on isotype stainings.

- [x] Tick this box to confirm that a figure exemplifying the gating strategy is provided in the Supplementary Information.
